# Quantification of Vortex Generation Due to Non-Equilibrium Electrokinetics at the Micro/Nanochannel Interface: Spectral Analysis

**DOI:** 10.3390/mi7070109

**Published:** 2016-06-27

**Authors:** Seung Jun Lee, Tae-Joon Jeon, Sun Min Kim, Daejoong Kim

**Affiliations:** 1 Department of Mechanical Engineering, Sogang University, Seoul 04107, Korea; seungjun@illinois.edu; 2 Department of Biological Engineering, Inha University, Incheon 22212, Korea; tjjeon@inha.ac.kr; 3 Department of Mechanical Engineering, Inha University, Incheon 22212, Korea

**Keywords:** Vortex generation, spectral analysis, concentration polarization, micro/nanochannel, micromixer, electrokinetics

## Abstract

We report on our investigation of a low Reynolds number non-equilibrium electrokinetic flow in a micro/nanochannel platform. Non-equilibrium electrokinetic phenomena include so-called concentration polarization in a moderate electric field and vortex formation in a high electric field. We conducted a spectral analysis of non-equilibrium electrokinetic vortices at a micro/nanochannel interface. We found that periodic vortices are formed while the frequency varies with the applied voltages and solution concentrations. At a frequency as high as 60 Hz, vortex generation was obtained with the strongest electric field and the lowest concentration. The power spectra show increasing frequency with increasing voltage or decreasing concentration. We expect that our spectral analysis results will be useful for micromixer developers in the micromachine research field.

## 1. Introduction 

With the improvement of micromachining technology over the past few years, the integration of nanoscale features has produced versatile microfluidic devices for various applications, such as sample preconcentration, biomolecule and DNA separation, and efficient filtration [1,2,3,4,5,6,7,8,9,10,11,12,13]. Recently, the ionic depletion-enrichment phenomenon in a micro-nanofluidic chip has attracted attention [2,5,6,7,9,10,14,15,16]. Researchers have reported that nanochannels (or nanopores) having nanometer dimensions exhibit unique ion perm-selectivity at low ionic strengths. These phenomena can be explained by an electric double layer overlap, since the electric double layer thickness is comparable to the nanochannel dimension [10,12,17,18,19,20]. In such a case, only selective ions can pass through the nanochannel. This ion selectivity results in so-called concentration polarization (CP) [5,6,7,14,21].

Non-equilibrium electrokinetic phenomena include CP and a nonlinear current–voltage relation across nanoporous membranes [2,6,10,14,16]. Concentration polarization refers to the creation of separate regions of enriched and depleted ion concentrations, respectively, upon the application of an electric field across micro/nanochannel interfaces. An interesting behavior that accompanies this concentration polarization is that local electrokinetic responses can be greatly amplified, especially in the ion depletion zone. As the CP zone widens in the depletion zone, a so-called depletion shock can occur [11,20,22,23,24,25]. This is possible when ions with low mobility travel in the same direction as the bulk flow toward the reservoir region, or when ions with high ion mobility move away from the depletion region. Applied voltages can be coupled with heterogeneous electric properties—in particular, gradients of ionic conductivity—to generate electric body forces in the bulk liquid. The depletion shocks result in a steep change in the concentration over a very short distance. These large concentration gradients in the background electrolyte create high electric field gradients. These strong electric field gradients at the ion depletion zone result in instability and consequently form strong vortices, which are useful in micromixing. Typical fluid flows in a microchannel have low Reynolds numbers since inertial forces were strongly damped by viscous forces [26,27]. Micromixing is, thus, one of the difficult problems in micromachine technology. We believe that non-equilibrium electrokinetic vortices can be efficiently utilized in micromixers.

There have been several experimental studies related to vortex generation induced by non-equilibrium electrokinetics in micro/nano fluidic mixing systems. Pu et al. first reported the visualization of CP near micro/nanochannel interfaces and showed a qualitative model of CP [19]. Han et al. first visualized strong vortex structures generated by the aforementioned mechanism [5,6,14,21]. Santiago et al. presented theoretical and experimental approaches to explain CP and vortex generation [10,11,26]. Kim et al. proposed a U-shaped microfluidic device utilizing vortices near micro/nanochannel interfaces to enable mixing [4]. They demonstrated the application of this type of mixer as an efficient micromixing technique. Song et al. described a quantitative study on a passive polydimethylsiloxane (PDMS) microfluidic mixer using a vortex index [28]. Lee and Kim achieved millisecond-order rapid mixing in their microfluidic device using vortices induced by non-equilibrium electrokinetics [9]. They also conducted a parametric study of active microfluidic mixing. Their results show that the mixing performance is greater with a higher applied voltage and a lower ion concentration. The mixing characteristics were quantified in terms of a mixing index and mixing time. Rubinstein et al. suggested similar non-equilibrium electrokinetic flow on the surface of perm-selective membranes [22]. 

We present spectral analysis results for non-equilibrium electrokinetic flows at micro/nanochannel interfaces. One related study is Posner et al. [26] (although the mechanism of vortex generation is different). They reported temporal power spectra with time-delay phase maps of the vortices in a T-shaped single microchannel, and analyzed the low Reynolds number flow with an electric Rayleigh number. In our spectral analysis at a micro/nanochannel interface, we measured the frequency under various conditions of electric fields and solution concentrations. The electric field affects the electric body forces inside a microchannel. The concentration of a working solution is related to the dimensions of an electric double layer. We expect that our results can contribute to an understanding of unstable electrokinetic flows and active microfluidic mixing. 

## 2. Experimental 

**Figure 1:**
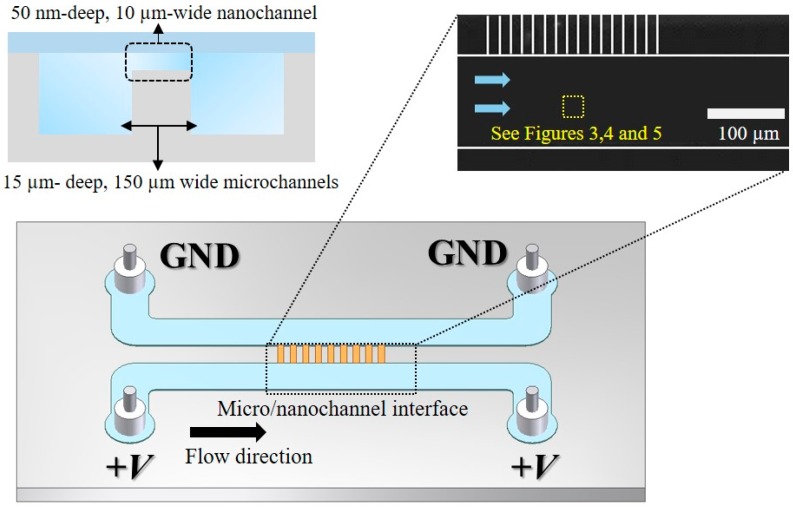
Schematic of the micro/nano channel device and the probing point for spectral analysis. The microchannel is 15 µm deep, 150 µm wide, and 1.5 cm long. The nanochannel is 10 µm wide and 50 nm deep. Inset: microscopic image of the fabricated microchannel and nanochannel.

The non-equilibrium electrokinetic microfluidic device considered in this study is comprised of double U-shaped microchannels and a set of nanochannels across the two sections of this microchannel, as shown in Figure 1. The microchannel has a single stream and there is short-circuited flow through nanochannels. The amount of fluid flow is expected to be negligible through the nanochannels because of high flow resistance. The purpose of these nanochannels is to create CP or vortex generation around the micro/nanochannel interface. We fabricated this device using a standard microelectromechanical systems (MEMS) process. We patterned the microchannels and nanochannels on a silicon substrate with photolithography, and etched these channels using a two-step reactive ion etching (RIE) process. For electrical insulation, we deposited SiO_2_ onto the substrate using a thermal oxidation process. Finally, we bonded a glass wafer to this patterned silicon substrate for sealing and visualization purposes. Mao and Han studied the temperatures of bonding required to maintain nanochannels while avoiding nanochannel collapse [29].

The microchannel is 15 µm deep, 150 µm wide, and has an overall length of approximately 1.5 cm. The distance between two microchannel sections, across which the set of eight nanochannels is located, is 50 µm. This distance, thus, coincides with the nanochannel length. All of the nanochannels are 50 nm deep, 10 µm wide, and have 10 µm spacing. The microfluidic device has four reservoirs at the end of two parallel microchannels (see Figure 1). Direct current (DC) electrical potentials were applied through submerged platinum wire electrodes in the electrolyte solutions at the reservoirs. We prepared various concentrations of potassium chloride (KCl) solution as the electrolyte solution and a small quantity of Rhodamine B (~0.3 µM) that was contained in an electrolyte solution. We were able to observe non-equilibrium vortex flow patterns in micro/nanochannel devices using inverted epifluorescent microscopy (IX-51, Olympus, New York, NY, USA) with a 20×, NA = 0.4 objective lens in a dark room. A charge-coupled device (CCD) camera (Coolsnap, Photometrics, Tucson, AZ, USA) connected to the microscope was used to take digital images of mixing patterns and transfer the visualization data to a personal computer (PC). We used a commercial power supply (IT 6834, Itech, Nanjing, China) to deliver electrical power to the microfluidic device through platinum wires submerged in the reservoirs. 

In the first step of the experiments, we completely filled the microchannels with KCl solution after the initial flushing steps with deionized water. Then, we applied DC electric potentials across the microfluidic device and observed the micro/nanochannel interfaces. By using the CCD camera, we recorded 10,000 frame images (the limit of our current setup in a single experiment) of vortex formation in 20 s at a frame rate of 500 frames/s. In our previous study, we calculated the millisecond-order mixing time for a similar micro-mixer design based on the same vortex generation mechanisms [9]. The frame rate of 500 frames/s was adequate for the time scale of the flow phenomena. For sufficient statistics, we repeated the recording eight times for both experimental conditions: the applied electric field and the concentration of the KCl solution.

## 3. Results and Discussion 

**Figure 2:**
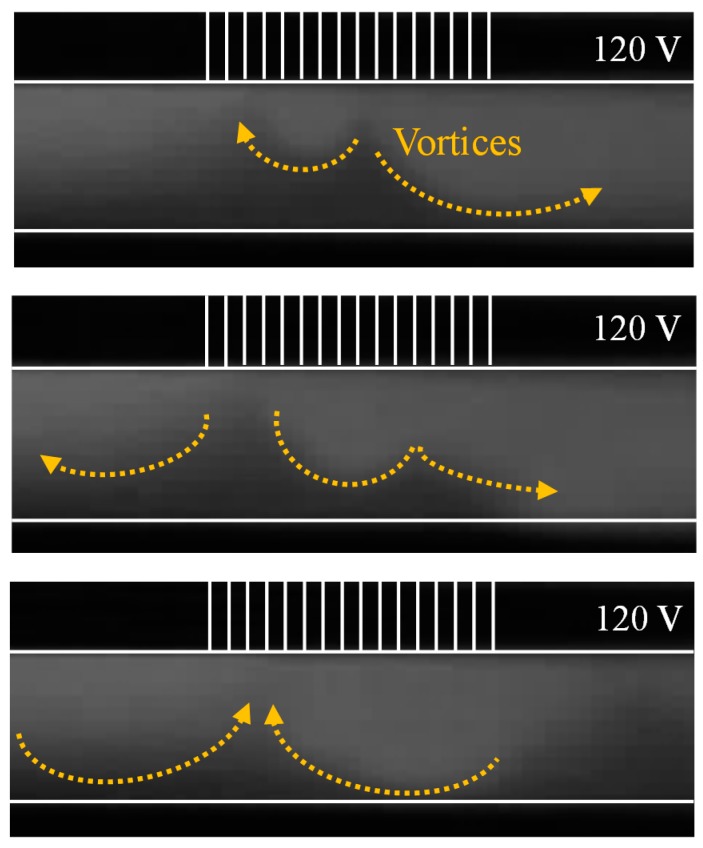
Images of vortex generation near the micro/nanochannel interface.

Figure 2 shows the visualized images of non-equilibrium electrokinetic vortices in the microfluidic device under applied electric fields. We obtained the time sequence of the fluorescent intensity field for all the experimental conditions. We normalized the digitalized intensity values in each pixel from 0 to 1 as follows:

(1) $$I_{\text{norm}}=\left|\frac{I-I_{\text{min}}}{I_{\text{max}}-I_{\text{min}}}\right|$$

where *I*_norm_ is the normalized value in each pixel; *I* is the measured fluorescent intensity in each pixel; and *I*_max_ and *I*_min_ are the bright-field and dark-field intensities, respectively. First, we subtracted the background noise from the images and we set a non-dye area as zero intensity with *I*_min_. We subsequently applied Equation (1) to our focused area for further analysis. Figure 2 shows three images taken at different time frames under the same experimental conditions. Vortices are found at the micro/nanochannel interface upon the application of an electric voltage of 120 V. The concentration of the working solution (KCl) is 0.5 mM. The Reynolds number inside the microfluidic channel is approximately 0.046, which was calculated using the hydraulic diameter and the flow rate. All experiments were performed with this fixed Reynolds number to analyze only the electrokinetic effects on the vortex formation in this device.

We selected the region of interest in the microchannel as the probing point for our spectral analysis (see Figure 1). This region is located near the micro/nanochannel interface along the center line of the microchannel. The probing point corresponds to a two-by-two square matrix of the normalized fluorescent intensities. We averaged these four intensity values to obtain a single representative value at each time frame. This representative fluorescent intensity at this probing point fluctuates significantly when vortices were formed during our experiments. We conducted the spectral analysis based on the time sequence of the representative fluorescent intensity value. 

**Figure 3:**
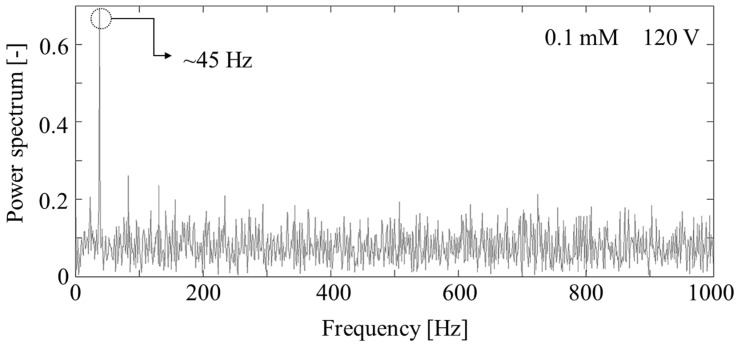
Example of the power spectrum.

In our spectral analysis, we used about 80,000 frames of vortex images around the micro/nanochannel interface. Figure 3 shows one example spectrum exhibiting a single peak near 50 Hz. This means that periodic vortices were formed around 45 Hz when we applied 120 V with 0.1 mM KCl solution. We performed the same type of spectral analysis for other experimental conditions.

**Figure 4:**
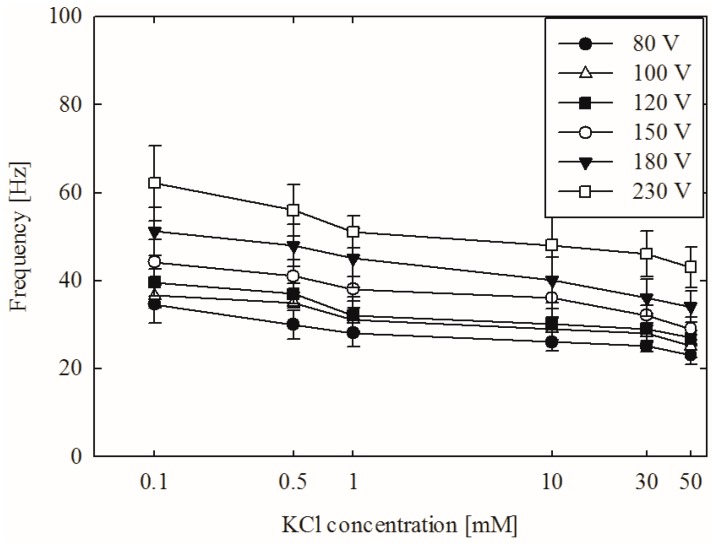
Spectral analysis results with different applied voltages.

**Figure 5:**
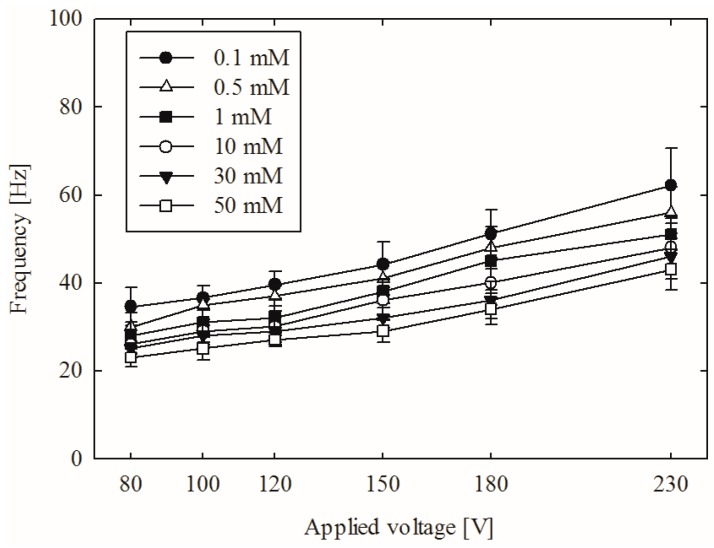
Spectral analysis results with different solution concentrations.

Figure 4 and Figure 5 show the power spectra under various voltage and concentration conditions. The frequency ranged from 29 to 60 Hz in our experiments with a voltage range of 80 to 230 V and a concentration of 0.1 to 50 mM. We established the periodic behavior of non-equilibrium electrokinetic vortices, which has not previously been reported in the literature. The maximum vortex frequency is 60 Hz at 230 V and 0.1 mM. The minimum vortex frequency is 29 Hz at 80 V and 50 mM.

Figure 4 shows the effect of different applied voltages on the frequency of vortex generation. A higher electric potential results in a higher vortex formation frequency in a system with a fixed KCl concentration. This indicates a stronger vortex formation in a stronger electric field. Kim et al. reported the relation between applied voltage and vortex formation by using a similar microfluidic mixer [4]. A stronger electric field generates a stronger electroosmotic flow inside the microfluidic chip, and the resulting electric body force enhances the collapsing of the CP effect. Upon the collapse of the concentration polarization effect, an abrupt ionic flux through the nanochannels is generated and it leads to unstable microflows near the micro/nanochannel interface. 

Figure 5 shows the relation between the concentration of the solution and the vortex formation frequency. A higher concentration of the solution decreases the size of the electric double layer on the nanochannels. This decrease lowers the ion perm-selectivity of the nanochannel (see Introduction for a detailed explanation). The lowered ion selectivity consequently mitigates the CP effect, thereby weakening vortex formation. The decreased size of the electric double layer is closely related to the so-called overlimiting current, which is an important factor in vortex formation. The thickness of an electric double layer depends on the corresponding electrolyte solution: the thicker the electrical double layer (EDL), the better the ion selectivity in a nanochannel, leading to faster and easier generation of a vortex. Moreover, the velocity of electroosmotic flow depends on electric fields in the channels. Therefore, higher voltage applied between electrodes with the same distance may increase the velocity of electro-osmotic flow, thus affecting the frequency of vortex generation. Our experimental spectral analysis observations are consistent with those reported in previous research (e.g., [4,9]). 

## 4. Conclusions 

We performed spectral analysis with a micro/nanochannel platform based on non-equilibrium electrokinetics. The collapse of the concentration polarization effect results in strong vortices near the micro/nanochannel interface, albeit at a low Reynolds number. We calculated the temporal power spectra of vortex generation and investigated the relation between frequency and two major operation parameters: the applied voltage and the solution concentration. The spectra show the periodic nature of non-equilibrium electrokinetic vortices. Higher frequency vortices are formed either with a stronger electric field or with a lower solution concentration. We believe that our results relating to the frequency of non-equilibrium electrokinetic vortices can be useful in future micromixer research and development.
